# Crystal structure and Hirshfeld surface analysis of (*E*)-4-chloro-*N*-{2-[2-(4-nitro­benzyl­idene)hydrazin-1-yl]-2-oxoeth­yl}benzene­sulfonamide *N*,*N*-di­methyl­formamide monosolvate

**DOI:** 10.1107/S205698901800292X

**Published:** 2018-02-23

**Authors:** H. Purandara, S. Foro, B. Thimme Gowda

**Affiliations:** a Department of Chemistry, Mangalore University, Mangalagangotri 574 199, Mangalore, India; b Department of Chemistry, Sri Dharmasthala Manjunatheshwara College (Autonomous), Ujire 574 240, India; c Institute of Materials Science, Darmstadt University of Technology, Alarich-Weiss-Str. 2, D-64287, Darmstadt, Germany; d Karnataka State Rural Development and Panchayat Raj University, Gadag 582 101, Karnataka, India

**Keywords:** crystal structure, hydrazone, inter­molecular hydrogen bonds, inversion dimers, Hirshfeld surface analysis

## Abstract

The asymmetric unit of the title compound, C_15_H_13_ClN_4_O_5_S·C_3_H_7_NO, contains one mol­ecule each of the Schiff base and the solvent di­methyl­formamide. The hydrazone group adopts an *E* configuration about the C=N bond. The dihedral angle between the two aromatic rings is 86.58 (2)°. In the crystal, pairs of N—H⋯O hydrogen bonds between centrosymmetrically related mol­ecules generates rings with an *R*_2_^2^(10) graph-set motif. The dimers are further linked *via* N—H⋯O and C—H⋯O hydrogen bonds, leading to the formation of *R*_3_^3^(11) ring motifs. C—H⋯π inter­actions are also observed. The inter­molecular inter­actions in the crystal structure were qu­anti­fied and analysed using Hirshfeld surface analysis, which indicates that the most significant contacts in packing are O⋯H/H⋯O (31.3%), followed by H⋯H (25.4%) and C⋯H/H⋯C (13.0%).

## Abstract 2

Reaction of *N*-(4-chloro­benzene­sulfon­yl)glycinyl hydrazide with 4-nitro­benzaldehyde gives the *N*,*N*-di­methyl­formamide monosolvated *N*-acyl­hydrazone derivative, (*E*)-*N*-{2-[2-(4-nitro­benzyl­idene)- hydrazine-1-yl]-2-oxoeth­yl}-4-χhloro­benzene­sulfonamide. Rings of 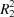
(10) and 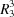
(11) graph-set motifs are formed in the crystal structure by N—H⋯O and C—H⋯O hydrogen bonds. The two-dimensional fingerprint (FP) plots for significant inter­molecular inter­actions indicate that the greatest contribution is from the O⋯H/H⋯O contacts (31.3%), corresponding to N⋯H⋯O/C⋯H⋯O inter­actions.

## Chemical context   

Supra­molecular chemistry is based upon non-covalent inter­actions such as hydrogen bonding, π–π stacking and van der Waals inter­actions (Beatty *et al.*, 2003 ▸; Biradha *et al.*, 2003 ▸; Aakeröy & Beatty, 2001 ▸). The presence of strong hydrogen-bond donors and acceptors on the mol­ecular periphery results in cross-linking of mol­ecules *via* strong hydrogen bonds into dimers, rings, chains and other hydrogen-bonded motifs. The acidity of the C—H donor group determines the strength of C—H⋯O inter­actions (Purandara *et al.*, 2017*a* ▸,*b* ▸). The study of C—H⋯O inter­actions in compounds containing chlorine atoms suggests that the more acidic the C—H hydrogen involved in a C—H⋯O inter­action, the stronger is the inter­action (Desiraju *et al.*, 1991 ▸). The presence of donors and acceptors make *N*-acyl­hydrazones important candidates for structural studies in this field. An attractive feature of hydrazones is their ability to form geometrical *E*/*Z* isomers because of the presence of the C=N double bond (Palla *et al.*, 1986 ▸) and conformational isomers because of a partly hindered rotation around the amide C—N bond. The nature and site of the substituents in the hydrazone moiety and hydrogen-bonding inter­actions decide the stereochemistry. In a continuation of our efforts to explore the effect of substit­uents on the structures of *N*-acyl­hydrazone derivatives, we report herein the synthesis, crystal structure and Hirshfeld analysis of the title compound, (*E*)-4-chloro-*N*-{2-[2-(4-nitro­benzyl­idene)hydrazin-1-yl]-2-oxoeth­yl}benzene­sulfonamide *N*,*N*-di­methyl­formamide monosolvate.
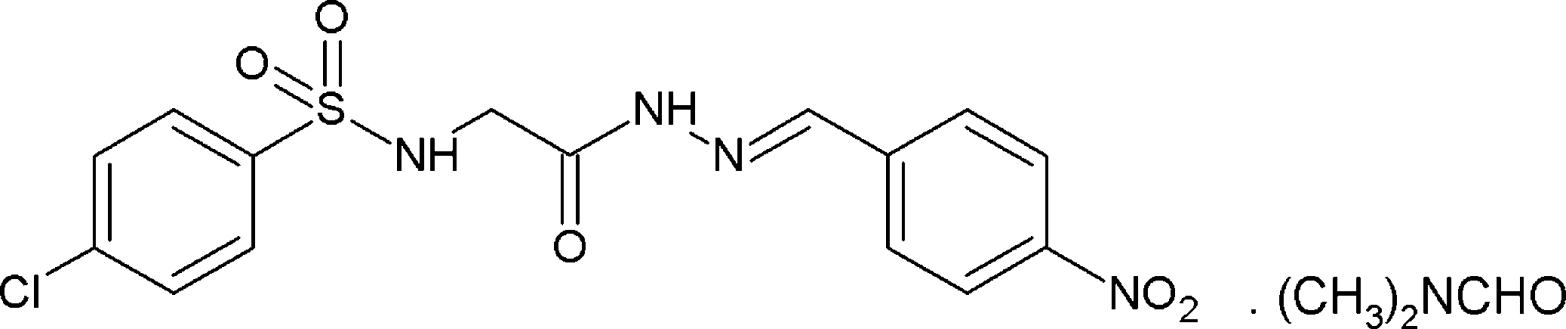


## Structural commentary   

**Figure 1:**
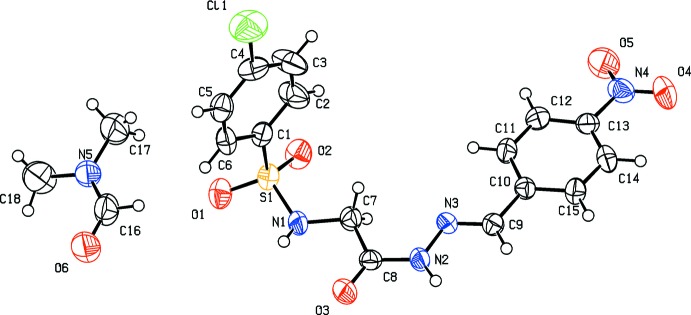
The mol­ecular structure of the title compound with displacement ellipsoids drawn at the 50% probability level.

The asymmetric unit of the title compound (Fig. 1 ▸) contains one mol­ecule each of the hydrazone and the solvent di­methyl­formamide (DMF). The mol­ecule displays an *E* configuration about the C=N bond. The conformations of the N—H, C—H and C=O bonds in the hydazone portion of the mol­ecule are *syn* to each other, whereas the C=O and N—H bonds in the glycinyl segment are *anti* to each other. The C8=O3 and C9=N3 bond lengths of 1.217 (6) and 1.274 (6) Å, respectively, confirm their double-bond character. The C8—N2 and N2—N3 bond distances [1.357 (7) and 1.374 (6) Å, respectively] are shorter than normal bond lengths as a result of delocalization of the π-electron density. The mol­ecule is twisted at N1—C7 with an S1—N1—C7—C8 torsion angle of 166.5 (4)°. The other central part of the mol­ecule is almost linear with C7—C8—N2—N3, C8—N2—N3—C9 and N2—N3—C9—C10 torsion angles of −1.6 (7), −179.7 (5) and 177.9 (4)°, respectively. The orientations of the sulfonamide group with respect to the attached phenyl ring is given by the torsion angles of C2—C1—S1—N1 = 98.1 (5)° and C6—C1—S1—N1 = −80.2 (5)°, while that of the hydrazone group with the attached phenyl ring by the torsion angles of C11—C10—C9—N3 = 1.6 (8)° and C15—C10—C9—N3 = −177.4 (5)°. The dihedral angle between the sulfonyl benzene ring and the mean plane through the SO_2_—NH—CH_2_—CO segment is 82.653 (18)°, while that between the C10–C15 phenyl ring and the mean plane through the C9—N3—N2—CO segment is 4.44 (3)°. The dihedral angle between the two aromatic rings is 86.58 (2)°. The C1–C6 and C10–C15 benzene rings are inclined to the mean plane of the central part of the hydrazone mol­ecule [O3/N1–N3/C7–C9; maximum deviation of 0.026 (6) Å for C7] by 86.4 (3) and 4.5 (3)°, respectively.

## Supra­molecular features   

**Figure 2:**
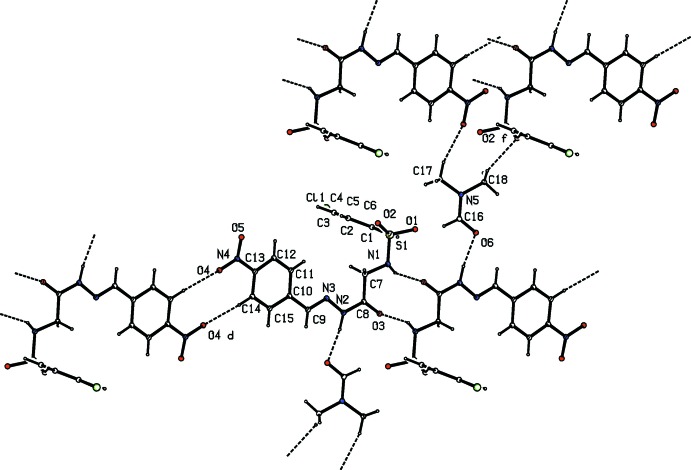
The hydrogen-bonding pattern (dashed lines) in the title compound.

**Figure 3:**
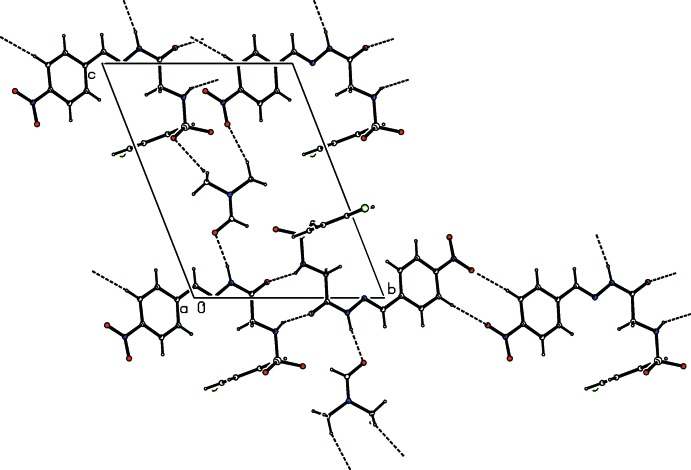
The mol­ecular packing of the title compound, with hydrogen bonding shown as dashed lines.

**Figure 4:**
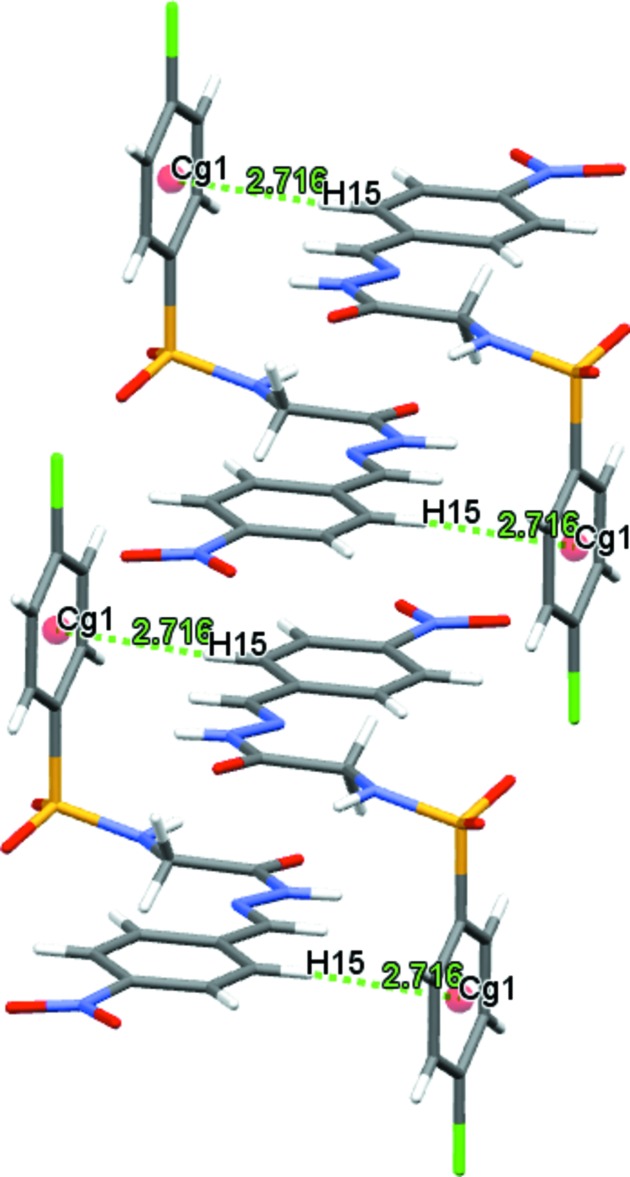
The C—H⋯π inter­actions (green dotted lines) observed in the structure of the title compound.

The hydrazone and solvent mol­ecules are connected *via* N—H⋯O and C—H⋯O hydrogen bonds, generating rings with an 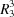
(11) graph-set motif (Table 1 ▸, Fig. 2 ▸). These bimolecular units are then linked by pairs of N—H⋯O hydrogen bonds, resulting in inversion dimers forming an 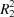
(10) ring motif. A pair of N—H⋯O hydrogen bonds connecting the sulfonamide H-atom of one mol­ecule with carbonyl O atom of another mol­ecule generates an 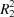
(10) ring, forming inversion dimers. The dimers are then linked *via* N—H⋯O and C—H⋯O hydrogen bonds, leading to the formation of 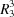
(11) ring motifs. These rings are further extended by two C—H⋯O hydrogen bonds, one involving a methyl hydrogen atom of the solvent mol­ecule (H18*B*) and the sulfonyl oxygen atom (O2) forming 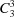
(18) chains along the *c* axis, and the other involving an aromatic C—H (H14) and the nitro O4 atom, giving rise to inversion dimers with an 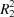
(10) graph-set motif (Fig. 3 ▸). In addition, the hydrazone mol­ecule is involved in C—H⋯π inter­actions (Fig. 4 ▸, Table 1 ▸). The hydrogen-bonding pattern in the title compound is similar to that observed in (*E*)-4-methyl-*N*-{2-[2-(4-nitro­benz­yl­idene)hydrazin-1-yl]-2-oxoeth­yl}benzene­sulfonamide *N*,*N*-di­meth­yl­form­amide monosolvate (Purandara *et al.*, 2017*a* ▸).

## Hirshfield Surface analysis   

*CrystalExplorer3.1* (Wolff *et al.*, 2012 ▸) was used to generate the mol­ecular Hirshfeld surfaces (*d*_norm_, electrostatic potential and curvedness) to analyse the close contacts in the title compound. The electrostatic potentials were calculated using *TONTO* (Spackman *et al.*, 2008 ▸; Jayatilaka *et al.*, 2005 ▸) integrated within *CrystalExplorer*. The mol­ecular Hirshfeld surfaces were generated using a standard (high) surface resolution with the 3D *d*_norm_ surfaces mapped over a fixed colour scale of −0.5849 to 1.3948. The curvedness was mapped in the colour range of −4.0 to 0.4. The electrostatic potentials were mapped on Hirshfeld surfaces using the STO-3G basis set at the Hartree–Fock level theory over a range ±0.1au.

**Figure 5:**
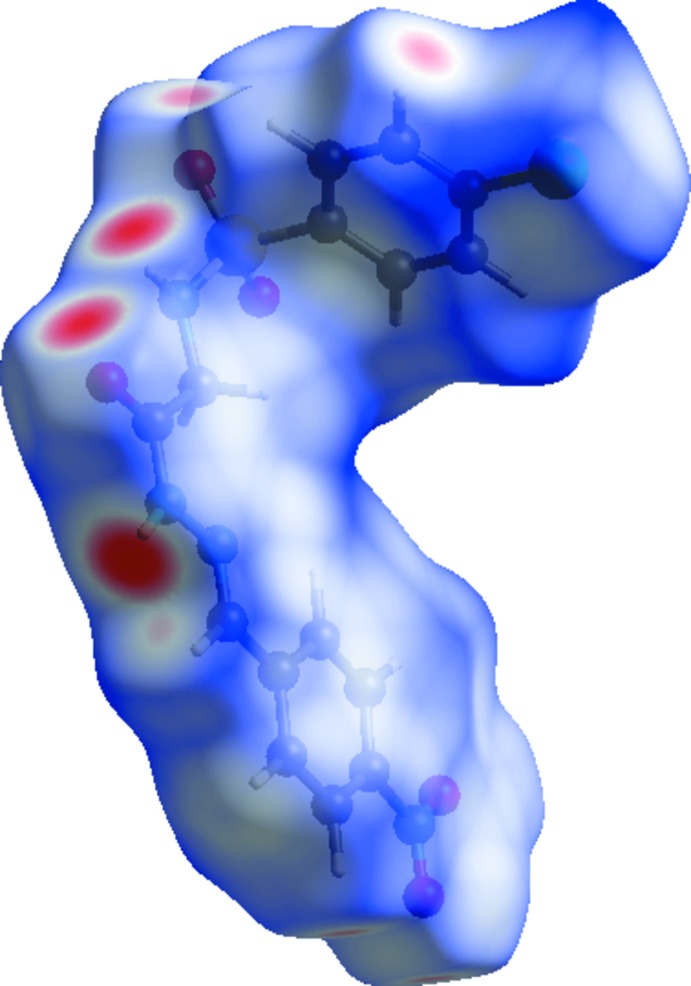
View of the Hirshfeld surface mapped over *d*_norm_.

**Figure 6:**
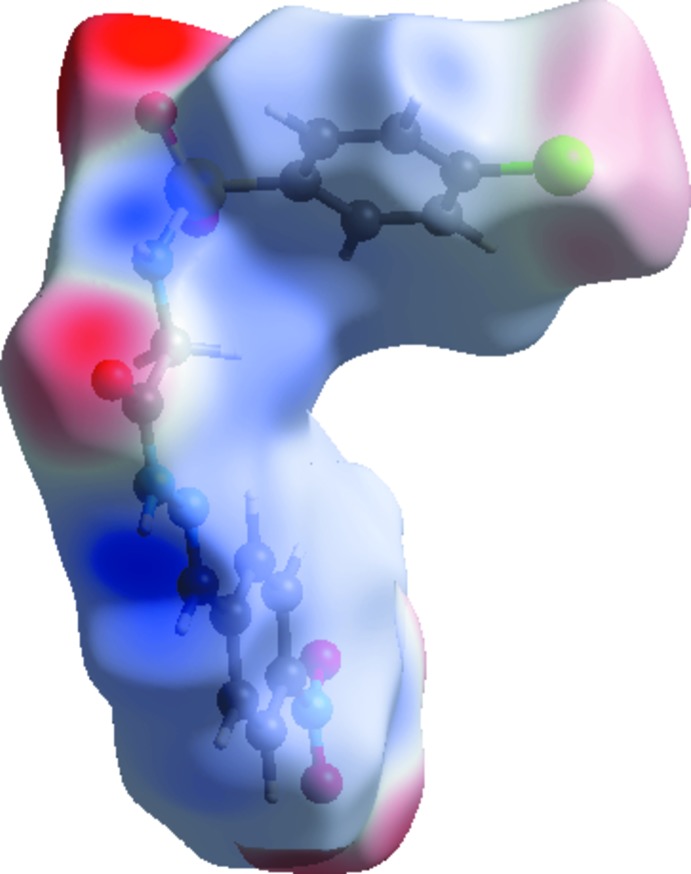
View of the Hirshfeld surface mapped over the electrostatic potential.

**Figure 7:**
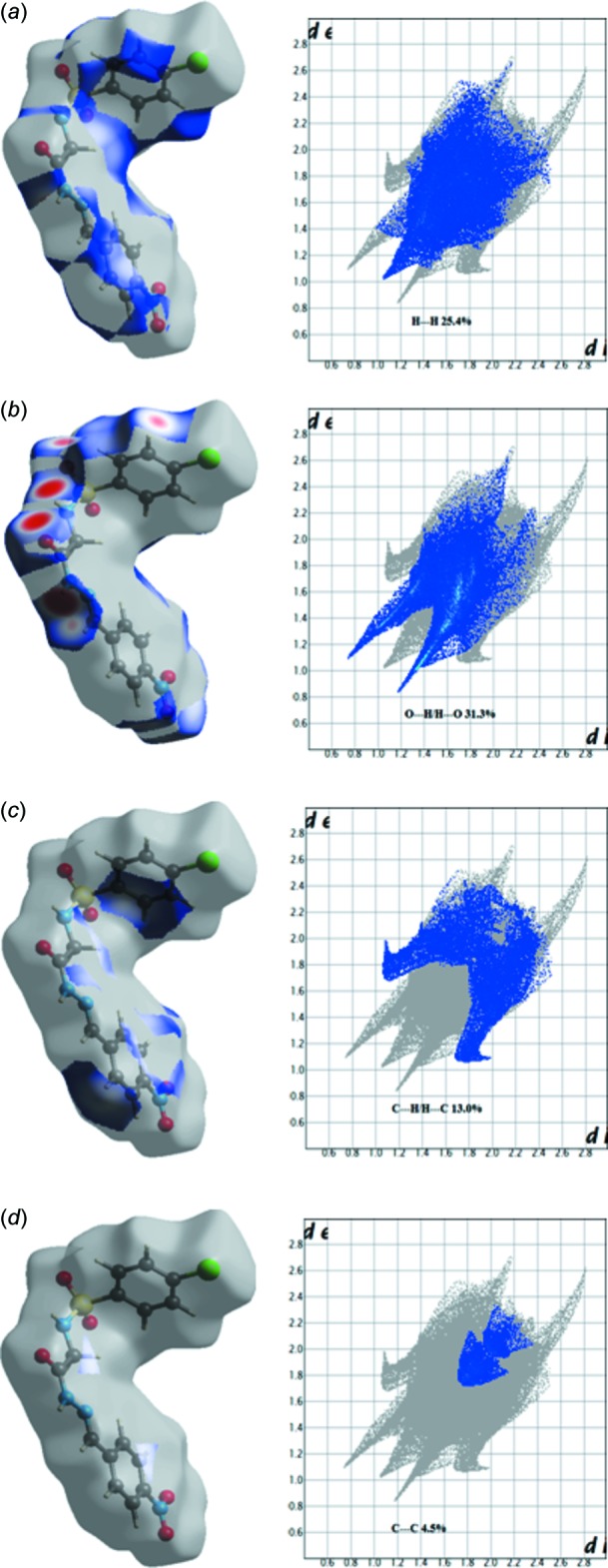
The two-dimensional fingerprint (FP) plot for the title compound, delineated into (*a*) O⋯H/H⋯O, (*b*) H⋯H, (*c*) C⋯H and (*d*) C⋯C inter­actions; *d*_norm_ surfaces for each plot, indicating the relevant surface patches associated with the specific contacts, are shown on the right.

**Figure 8:**
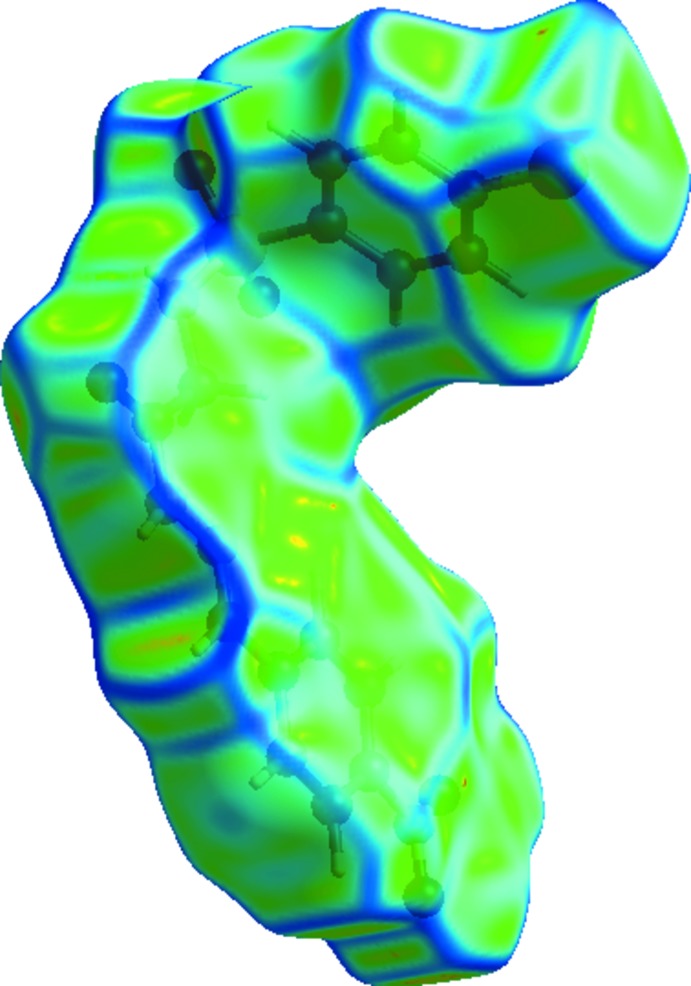
View of the Hirshfeld surface mapped over curvedness.

In the Hirshfeld surfaces mapped over *d*_norm_ (Fig. 5 ▸), the strong N—H⋯O inter­actions can be observed as bright-red spots between oxygen (O) and hydrogen (H) atoms. These inter­actions are further confirmed by Hirshfeld surfaces mapped over the electrostatic potential (Fig. 6 ▸), showing the negative potential around the oxygen atoms as light-red clouds and the positive potential around hydrogen atoms as light-blue clouds. The two-dimensional fingerprint (FP) plots for significant inter­molecular inter­actions are illustrated in Fig. 7 ▸. The greatest contribution from the O⋯H/H⋯O contacts is 31.3%, corresponding to N—H⋯O/C—H⋯O inter­actions, is represented by a pair of sharp spikes characteristic of a strong hydrogen-bonding inter­action having *d*_e_ + *d*_i_ values of about 1.8 and 2.0 Å (Fig. 7 ▸ *b*). The H⋯H inter­actions appear as the largest region of the fingerprint plot with a high concentration in the middle region, shown in light blue, at *d*_e_ = *d*_i_ ∼1.4 Å (Fig. 7 ▸ *a*) with an overall contribution to the Hirshfeld surfaces of 25.4%. The C⋯H contacts, which refer to C—H⋯π inter­actions, contribute 13.0% of the Hirshfeld surfaces. The presence of C—H⋯π inter­actions is indicated by the appearance of two broad spikes having almost same *d*_e_ + *d*_i_ 3.1 Å. The C⋯C contacts contribute 4.5% of the Hirshfeld surfaces, featuring two successive triangles with a minimum (*d*_e_ + *d*_i_) distance of ∼3.5 Å, which is greater than van der Waals separation, confirming the absence of π–π stacking inter­actions. This is also evident from the absence of flat regions in the Hirshfeld surface mapped over curvedness (Fig. 8 ▸).

## Synthesis and crystallization   

4-Chloro­benzene­sulfonyl chloride (0.01 mol) was added to glycine (0.02 mol) dissolved in an aqueous solution of potassium carbonate (0.06 mol, 50 ml). The reaction mixture was stirred at 373 K for 6 h, left overnight at room temperature, then filtered and treated with dilute hydro­chloric acid. The solid *N*-(4-chloro­benzene­sulfon­yl)glycine (**L1**) obtained was crystallized from aqueous ethanol. Sulfuric acid (0.5 ml) was added to **L1** (0.02 mol) dissolved in ethanol (30 ml) and the mixture was refluxed. The reaction mixture was monitored by TLC at regular inter­vals. After completion of the reaction, the reaction mixture was concentrated to remove the excess ethanol. The product, *N*-(4-chloro­benzene­sulfon­yl)glycine ethyl ester (**L2**) obtained was poured into water, neutralized with sodium bicarbonate and recrystallized from acetone. The pure **L2** (0.01 mol) was then added in small portions to a stirred solution of 99% hydrazine hydrate (10 ml) in 30 ml ethanol and the mixture was refluxed for 6 h. After cooling to room temperature, the resulting precipitate was filtered, washed with cold water and dried to obtain *N*-(4-chloro­benzene­sulfon­yl)glycinyl hydrazide (**L3**). A mixture of **L3** (0.01 mol) and 4-nitro­benzaldehyde (0.01 mol) in anhydrous methanol (30 ml) and two drops of glacial acetic acid was refluxed for 8h. After cooling, the precipitate was collected by vacuum filtration, washed with cold methanol and dried. It was recrystallized to a constant melting point from methanol (493–496 K).

The purity of the compound was checked by TLC and characterized by its IR spectrum. The characteristic absorptions observed are 3250.1, 1685.8, 1587.4, 1342.5 and 1166.9 cm^−1^ for the stretching bands of N—H, C=O, C=N, S=O asymmetric and S=O symmetric, respectively. ^1^H NMR (400 MHz, DMSO-*d*_6_, *δ* ppm): 3.68, 4.17 (2*d*, 2H, *J* = 5.68 Hz), 7.62–7.67 (*m*, 2H, Ar-H), 7.80–7.94 (*m*, 4H, Ar-H), 8.24–8.29 (*m*, 2H, Ar-H), 8.02 (*s*, 1H), 8.14 (*t*, 1H), 11.73, 11.75 (2*s*, 1H). ^13^C NMR (400 MHz, DMSO-*d*_6_, *δ* ppm): 43.26, 44.42, 123.94, 127.85, 128.53, 129.19, 137.23, 139.77, 141.47, 144.68, 147.75, 164.52, 169.34. Plate-like yellow single crystals of the title compound suitable for X-ray analysis were grown from its DMF solution by slow evaporation of the solvent.

## Refinement   

Crystal data, data collection and structure refinement details are summarized in Table 2 ▸. H atoms bonded to C atoms were positioned with idealized geometry, C—H = 0.93 (aromatic), 0.96 (meth­yl) or 0.97 Å (methyl­ene) and refined using a riding model with isotropic displacement parameters set at 1.2*U*_eq_(C, N) or 1.5*U*_eq_(C) for methyl H atoms.. The amino H atoms were freely refined with the N—H distances restrained to 0.86 (2) Å.

## Supplementary Material

Crystal structure: contains datablock(s) I. DOI: 10.1107/S205698901800292X/rz5227sup1.cif

Structure factors: contains datablock(s) I. DOI: 10.1107/S205698901800292X/rz5227Isup2.hkl

Supporting information file. DOI: 10.1107/S205698901800292X/rz5227Isup3.cml

CCDC reference: 1433601

Additional supporting information:  crystallographic information; 3D view; checkCIF report

## Figures and Tables

**Table 1:** Hydrogen-bond geometry (Å, °)

*D*—H⋯*A*	*D*—H	H⋯*A*	*D*⋯*A*	*D*—H⋯*A*
N1—H1*N*⋯O3^i^	0.85 (2)	2.18 (3)	2.976 (6)	155 (5)
N2—H2*N*⋯O6^i^	0.87 (2)	2.00 (2)	2.857 (6)	171 (5)
C5—H5⋯O2^ii^	0.93	2.47	3.357 (7)	159
C14—H14⋯O4^iii^	0.93	2.53	3.457 (7)	175
C16—H16*A*⋯O1	0.93	2.46	3.207 (8)	138
C18—H18*B*⋯O2^iv^	0.96	2.53	3.339 (8)	142
C15—H15⋯*Cg*1^v^	0.93	2.72	3.629 (7)	167

Symmetry codes: (i) 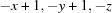; (ii) 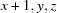; (iii) 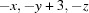; (iv) 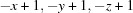; (v) 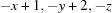.

**Table 2:** Experimental details

Crystal data	
Chemical formula	C_15_H_13_ClN_4_O_5_S·C_3_H_7_NO
*M*_r_	469.90
Crystal system, space group	Triclinic, *P* 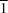
Temperature (K)	293
*a*, *b*, *c* (Å)	8.240 (1), 10.631 (1), 13.720 (2)
α, β, γ (°)	108.15 (1), 98.36 (1), 105.07 (1)
*V* (Å^3^)	1068.7 (2)
*Z*	2
Radiation type	Mo *K*α
μ (mm^−1^)	0.32
Crystal size (mm)	0.46 × 0.22 × 0.08

Data collection	
Diffractometer	Oxford Diffraction Xcalibur diffractometer with Sapphire CCD detector
Absorption correction	Multi-scan (*CrysAlis RED*; Oxford Diffraction, 2009 ▸)
*T*_min_, *T*_max_	0.866, 0.975
No. of measured, independent and observed [*I* > 2σ(*I*)] reflections	6809, 3847, 2623
*R*_int_	0.027
(sin θ/λ)_max_ (Å^−1^)	0.602

Refinement	
*R*[*F* ^2^ > 2σ(*F* ^2^)], *wR*(*F* ^2^), *S*	0.090, 0.167, 1.32
No. of reflections	3847
No. of parameters	288
No. of restraints	2
H-atom treatment	H atoms treated by a mixture of independent and constrained refinement
Δρ_max_, Δρ_min_ (e Å^−3^)	0.61, −0.43

Computer programs: *CrysAlis CCD* and *CrysAlis RED* (Oxford Diffraction, 2009 ▸), *SHELXS2013* (Sheldrick, 2008 ▸), *SHELXL2014* (Sheldrick, 2015 ▸), *Mercury* (Macrae *et al.*, 2008 ▸) and *PLATON* (Spek, 2015 ▸).

## supplementary crystallographic information

### Crystal data

**Table:**

C_15_H_13_ClN_4_O_5_S·C_3_H_7_NO	*Z* = 2
*M**_r_* = 469.90	*F*(000) = 488
Triclinic, *P*1	*D*_x_ = 1.460 Mg m^−^^3^
*a* = 8.240 (1) Å	Mo *K*α radiation, λ = 0.71073 Å
*b* = 10.631 (1) Å	Cell parameters from 1907 reflections
*c* = 13.720 (2) Å	θ = 2.6–28.0°
α = 108.15 (1)°	µ = 0.32 mm^−^^1^
β = 98.36 (1)°	*T* = 293 K
γ = 105.07 (1)°	Plate, yellow
*V* = 1068.7 (2) Å^3^	0.46 × 0.22 × 0.08 mm

### Data collection

**Table:**

Oxford Diffraction Xcalibur diffractometer with Sapphire CCD detector	2623 reflections with *I* > 2σ(*I*)
Radiation source: Enhance (Mo) X-ray Source	*R*_int_ = 0.027
Rotation method data acquisition using ω scans.	θ_max_ = 25.4°, θ_min_ = 2.6°
Absorption correction: multi-scan (CrysAlis RED; Oxford Diffraction, 2009)	*h* = −9→8
*T*_min_ = 0.866, *T*_max_ = 0.975	*k* = −12→12
6809 measured reflections	*l* = −14→16
3847 independent reflections	

### Refinement

**Table:**

Refinement on *F*^2^	2 restraints
Least-squares matrix: full	Hydrogen site location: mixed
*R*[*F*^2^ > 2σ(*F*^2^)] = 0.090	H atoms treated by a mixture of independent and constrained refinement
*wR*(*F*^2^) = 0.167	*w* = 1/[σ^2^(*F*_o_^2^) + 3.0287*P*] where *P* = (*F*_o_^2^ + 2*F*_c_^2^)/3
*S* = 1.32	(Δ/σ)_max_ < 0.001
3847 reflections	Δρ_max_ = 0.61 e Å^−^^3^
288 parameters	Δρ_min_ = −0.43 e Å^−^^3^

### Special details

**Table:**

Geometry. All esds (except the esd in the dihedral angle between two l.s. planes) are estimated using the full covariance matrix. The cell esds are taken into account individually in the estimation of esds in distances, angles and torsion angles; correlations between esds in cell parameters are only used when they are defined by crystal symmetry. An approximate (isotropic) treatment of cell esds is used for estimating esds involving l.s. planes.

### Fractional atomic coordinates and isotropic or equivalent isotropic displacement parameters (Å^2^)

**Table:**

	*x*	*y*	*z*	*U*_iso_*/*U*_eq_	
Cl1	1.2884 (2)	1.0818 (2)	0.38232 (19)	0.0962 (7)	
S1	0.52061 (19)	0.69212 (16)	0.26923 (12)	0.0430 (4)	
O1	0.5214 (5)	0.5704 (4)	0.2924 (3)	0.0570 (11)	
O2	0.4256 (5)	0.7784 (4)	0.3183 (3)	0.0554 (11)	
O3	0.3990 (5)	0.5834 (4)	−0.0674 (3)	0.0561 (11)	
O4	0.0376 (6)	1.5139 (5)	0.1175 (4)	0.0744 (14)	
O5	0.0718 (7)	1.4667 (5)	0.2589 (4)	0.0824 (16)	
N1	0.4475 (6)	0.6423 (5)	0.1438 (4)	0.0429 (11)	
H1N	0.482 (7)	0.581 (4)	0.103 (4)	0.051*	
N2	0.3264 (6)	0.7745 (5)	−0.0636 (4)	0.0420 (11)	
H2N	0.321 (7)	0.760 (6)	−0.1298 (19)	0.050*	
N3	0.2997 (5)	0.8933 (4)	−0.0024 (3)	0.0364 (10)	
N4	0.0771 (6)	1.4444 (5)	0.1669 (5)	0.0534 (13)	
C1	0.7391 (7)	0.8014 (6)	0.3018 (4)	0.0406 (13)	
C2	0.7777 (9)	0.9455 (7)	0.3399 (6)	0.069 (2)	
H2	0.6897	0.9846	0.3496	0.083*	
C3	0.9470 (9)	1.0308 (7)	0.3635 (6)	0.077 (2)	
H3	0.9738	1.1275	0.3882	0.092*	
C4	1.0758 (8)	0.9717 (7)	0.3501 (5)	0.0572 (17)	
C5	1.0392 (8)	0.8290 (7)	0.3112 (5)	0.0495 (15)	
H5	1.1276	0.7904	0.3015	0.059*	
C6	0.8695 (8)	0.7438 (6)	0.2866 (5)	0.0467 (15)	
H6	0.8429	0.6470	0.2598	0.056*	
C7	0.4001 (7)	0.7410 (6)	0.1009 (4)	0.0421 (13)	
H7A	0.4905	0.8313	0.1332	0.051*	
H7B	0.2936	0.7527	0.1181	0.051*	
C8	0.3754 (7)	0.6906 (5)	−0.0167 (4)	0.0386 (13)	
C9	0.2531 (7)	0.9679 (5)	−0.0504 (4)	0.0383 (13)	
H9	0.2425	0.9424	−0.1229	0.046*	
C10	0.2161 (6)	1.0935 (5)	0.0081 (4)	0.0355 (12)	
C11	0.2339 (8)	1.1382 (6)	0.1170 (4)	0.0483 (15)	
H11	0.2757	1.0895	0.1548	0.058*	
C12	0.1910 (8)	1.2531 (6)	0.1696 (5)	0.0506 (15)	
H12	0.2019	1.2819	0.2422	0.061*	
C13	0.1312 (7)	1.3247 (6)	0.1119 (4)	0.0408 (13)	
C14	0.1167 (7)	1.2873 (6)	0.0058 (5)	0.0467 (14)	
H14	0.0795	1.3390	−0.0309	0.056*	
C15	0.1584 (7)	1.1714 (6)	−0.0455 (5)	0.0450 (14)	
H15	0.1479	1.1442	−0.1180	0.054*	
O6	0.7001 (7)	0.2440 (5)	0.2779 (4)	0.0807 (16)	
N5	0.7345 (7)	0.4007 (5)	0.4406 (4)	0.0602 (14)	
C16	0.7123 (10)	0.3594 (8)	0.3368 (6)	0.069 (2)	
H16A	0.7055	0.4252	0.3062	0.083*	
C17	0.7557 (13)	0.5436 (8)	0.5057 (6)	0.104 (3)	
H17A	0.7493	0.5976	0.4616	0.156*	
H17B	0.8665	0.5839	0.5560	0.156*	
H17C	0.6654	0.5439	0.5427	0.156*	
C18	0.7419 (12)	0.3051 (8)	0.4940 (6)	0.091 (3)	
H18A	0.7043	0.2114	0.4429	0.136*	
H18B	0.6675	0.3117	0.5418	0.136*	
H18C	0.8589	0.3282	0.5330	0.136*	

### Atomic displacement parameters (Å^2^)

**Table:**

	*U*^11^	*U*^22^	*U*^33^	*U*^12^	*U*^13^	*U*^23^
Cl1	0.0548 (12)	0.0931 (16)	0.1126 (18)	0.0029 (11)	0.0042 (11)	0.0249 (13)
S1	0.0452 (8)	0.0514 (9)	0.0430 (8)	0.0233 (7)	0.0149 (7)	0.0232 (7)
O1	0.061 (3)	0.061 (3)	0.063 (3)	0.023 (2)	0.017 (2)	0.038 (2)
O2	0.053 (3)	0.072 (3)	0.056 (3)	0.037 (2)	0.025 (2)	0.023 (2)
O3	0.074 (3)	0.047 (3)	0.050 (3)	0.034 (2)	0.013 (2)	0.010 (2)
O4	0.090 (4)	0.059 (3)	0.090 (4)	0.048 (3)	0.021 (3)	0.030 (3)
O5	0.109 (4)	0.080 (4)	0.063 (3)	0.055 (3)	0.026 (3)	0.010 (3)
N1	0.052 (3)	0.040 (3)	0.043 (3)	0.027 (2)	0.009 (2)	0.015 (2)
N2	0.057 (3)	0.038 (3)	0.036 (3)	0.026 (2)	0.011 (2)	0.014 (2)
N3	0.040 (3)	0.031 (2)	0.039 (3)	0.014 (2)	0.009 (2)	0.010 (2)
N4	0.048 (3)	0.045 (3)	0.061 (4)	0.020 (3)	0.011 (3)	0.008 (3)
C1	0.044 (3)	0.049 (3)	0.036 (3)	0.024 (3)	0.012 (3)	0.018 (3)
C2	0.060 (4)	0.052 (4)	0.085 (5)	0.031 (4)	0.017 (4)	0.001 (4)
C3	0.054 (4)	0.041 (4)	0.105 (6)	0.009 (3)	0.014 (4)	−0.005 (4)
C4	0.044 (4)	0.062 (4)	0.048 (4)	0.007 (3)	0.003 (3)	0.009 (3)
C5	0.045 (4)	0.062 (4)	0.054 (4)	0.029 (3)	0.018 (3)	0.027 (3)
C6	0.057 (4)	0.048 (4)	0.053 (4)	0.030 (3)	0.021 (3)	0.027 (3)
C7	0.047 (3)	0.043 (3)	0.041 (3)	0.022 (3)	0.012 (3)	0.016 (3)
C8	0.034 (3)	0.034 (3)	0.040 (3)	0.010 (2)	0.003 (2)	0.007 (3)
C9	0.043 (3)	0.038 (3)	0.035 (3)	0.014 (3)	0.010 (2)	0.015 (3)
C10	0.032 (3)	0.034 (3)	0.042 (3)	0.009 (2)	0.008 (2)	0.016 (2)
C11	0.065 (4)	0.048 (4)	0.038 (3)	0.027 (3)	0.007 (3)	0.019 (3)
C12	0.065 (4)	0.048 (4)	0.035 (3)	0.022 (3)	0.005 (3)	0.010 (3)
C13	0.039 (3)	0.037 (3)	0.039 (3)	0.011 (3)	0.004 (3)	0.008 (3)
C14	0.050 (4)	0.040 (3)	0.061 (4)	0.020 (3)	0.015 (3)	0.027 (3)
C15	0.051 (4)	0.049 (4)	0.045 (3)	0.023 (3)	0.013 (3)	0.025 (3)
O6	0.135 (5)	0.075 (3)	0.045 (3)	0.056 (3)	0.028 (3)	0.017 (3)
N5	0.085 (4)	0.057 (3)	0.042 (3)	0.034 (3)	0.014 (3)	0.014 (3)
C16	0.100 (6)	0.075 (5)	0.052 (5)	0.051 (5)	0.022 (4)	0.030 (4)
C17	0.165 (9)	0.083 (6)	0.064 (5)	0.072 (6)	0.004 (5)	0.010 (5)
C18	0.145 (8)	0.077 (5)	0.056 (5)	0.032 (5)	0.034 (5)	0.030 (4)

### Geometric parameters (Å, º)

**Table:**

Cl1—C4	1.739 (6)	C7—H7A	0.9700
S1—O1	1.427 (4)	C7—H7B	0.9700
S1—O2	1.431 (4)	C9—C10	1.461 (7)
S1—N1	1.603 (5)	C9—H9	0.9300
S1—C1	1.773 (6)	C10—C15	1.390 (7)
O3—C8	1.217 (6)	C10—C11	1.392 (7)
O4—N4	1.217 (6)	C11—C12	1.374 (8)
O5—N4	1.221 (6)	C11—H11	0.9300
N1—C7	1.460 (6)	C12—C13	1.380 (7)
N1—H1N	0.854 (19)	C12—H12	0.9300
N2—C8	1.357 (7)	C13—C14	1.362 (8)
N2—N3	1.374 (6)	C14—C15	1.372 (7)
N2—H2N	0.865 (19)	C14—H14	0.9300
N3—C9	1.274 (6)	C15—H15	0.9300
N4—C13	1.477 (7)	O6—C16	1.210 (8)
C1—C6	1.379 (7)	N5—C16	1.322 (8)
C1—C2	1.386 (8)	N5—C18	1.434 (8)
C2—C3	1.379 (9)	N5—C17	1.452 (8)
C2—H2	0.9300	C16—H16A	0.9300
C3—C4	1.373 (9)	C17—H17A	0.9600
C3—H3	0.9300	C17—H17B	0.9600
C4—C5	1.375 (8)	C17—H17C	0.9600
C5—C6	1.380 (8)	C18—H18A	0.9600
C5—H5	0.9300	C18—H18B	0.9600
C6—H6	0.9300	C18—H18C	0.9600
C7—C8	1.496 (7)		
			
O1—S1—O2	120.2 (3)	O3—C8—C7	123.2 (5)
O1—S1—N1	107.3 (2)	N2—C8—C7	115.1 (5)
O2—S1—N1	107.0 (2)	N3—C9—C10	120.2 (5)
O1—S1—C1	107.6 (3)	N3—C9—H9	119.9
O2—S1—C1	106.8 (3)	C10—C9—H9	119.9
N1—S1—C1	107.4 (3)	C15—C10—C11	117.8 (5)
C7—N1—S1	118.6 (4)	C15—C10—C9	119.9 (5)
C7—N1—H1N	116 (4)	C11—C10—C9	122.2 (5)
S1—N1—H1N	118 (4)	C12—C11—C10	121.2 (5)
C8—N2—N3	119.1 (4)	C12—C11—H11	119.4
C8—N2—H2N	122 (4)	C10—C11—H11	119.4
N3—N2—H2N	118 (4)	C11—C12—C13	118.4 (5)
C9—N3—N2	116.6 (4)	C11—C12—H12	120.8
O4—N4—O5	123.6 (5)	C13—C12—H12	120.8
O4—N4—C13	118.4 (6)	C14—C13—C12	122.5 (5)
O5—N4—C13	118.0 (5)	C14—C13—N4	118.7 (5)
C6—C1—C2	120.0 (6)	C12—C13—N4	118.8 (5)
C6—C1—S1	120.4 (4)	C13—C14—C15	118.3 (5)
C2—C1—S1	119.6 (4)	C13—C14—H14	120.9
C3—C2—C1	119.7 (6)	C15—C14—H14	120.9
C3—C2—H2	120.1	C14—C15—C10	121.8 (5)
C1—C2—H2	120.1	C14—C15—H15	119.1
C4—C3—C2	119.5 (6)	C10—C15—H15	119.1
C4—C3—H3	120.2	C16—N5—C18	120.7 (6)
C2—C3—H3	120.2	C16—N5—C17	122.2 (6)
C3—C4—C5	121.4 (6)	C18—N5—C17	117.1 (6)
C3—C4—Cl1	118.5 (5)	O6—C16—N5	126.0 (7)
C5—C4—Cl1	120.1 (5)	O6—C16—H16A	117.0
C4—C5—C6	119.0 (5)	N5—C16—H16A	117.0
C4—C5—H5	120.5	N5—C17—H17A	109.5
C6—C5—H5	120.5	N5—C17—H17B	109.5
C1—C6—C5	120.3 (6)	H17A—C17—H17B	109.5
C1—C6—H6	119.8	N5—C17—H17C	109.5
C5—C6—H6	119.8	H17A—C17—H17C	109.5
N1—C7—C8	111.2 (4)	H17B—C17—H17C	109.5
N1—C7—H7A	109.4	N5—C18—H18A	109.5
C8—C7—H7A	109.4	N5—C18—H18B	109.5
N1—C7—H7B	109.4	H18A—C18—H18B	109.5
C8—C7—H7B	109.4	N5—C18—H18C	109.5
H7A—C7—H7B	108.0	H18A—C18—H18C	109.5
O3—C8—N2	121.7 (5)	H18B—C18—H18C	109.5
			
O1—S1—N1—C7	167.8 (4)	N3—N2—C8—C7	−1.6 (7)
O2—S1—N1—C7	37.6 (5)	N1—C7—C8—O3	−2.5 (8)
C1—S1—N1—C7	−76.7 (5)	N1—C7—C8—N2	178.3 (5)
C8—N2—N3—C9	−179.7 (5)	N2—N3—C9—C10	177.9 (4)
O1—S1—C1—C6	35.0 (5)	N3—C9—C10—C15	−177.4 (5)
O2—S1—C1—C6	165.3 (4)	N3—C9—C10—C11	1.6 (8)
N1—S1—C1—C6	−80.2 (5)	C15—C10—C11—C12	2.0 (9)
O1—S1—C1—C2	−146.7 (5)	C9—C10—C11—C12	−177.1 (5)
O2—S1—C1—C2	−16.4 (6)	C10—C11—C12—C13	−0.7 (9)
N1—S1—C1—C2	98.1 (5)	C11—C12—C13—C14	−1.3 (9)
C6—C1—C2—C3	−0.6 (10)	C11—C12—C13—N4	177.2 (5)
S1—C1—C2—C3	−178.8 (6)	O4—N4—C13—C14	−7.1 (8)
C1—C2—C3—C4	−0.8 (12)	O5—N4—C13—C14	171.7 (6)
C2—C3—C4—C5	1.6 (12)	O4—N4—C13—C12	174.3 (6)
C2—C3—C4—Cl1	−179.1 (6)	O5—N4—C13—C12	−7.0 (8)
C3—C4—C5—C6	−1.0 (10)	C12—C13—C14—C15	2.0 (9)
Cl1—C4—C5—C6	179.7 (5)	N4—C13—C14—C15	−176.6 (5)
C2—C1—C6—C5	1.2 (9)	C13—C14—C15—C10	−0.7 (8)
S1—C1—C6—C5	179.5 (4)	C11—C10—C15—C14	−1.3 (8)
C4—C5—C6—C1	−0.5 (9)	C9—C10—C15—C14	177.8 (5)
S1—N1—C7—C8	166.5 (4)	C18—N5—C16—O6	−1.6 (12)
N3—N2—C8—O3	179.2 (5)	C17—N5—C16—O6	177.1 (8)

### Hydrogen-bond geometry (Å, º)

**Table:**

*D*—H···*A*	*D*—H	H···*A*	*D*···*A*	*D*—H···*A*
N1—H1*N*···O3^i^	0.85 (2)	2.18 (3)	2.976 (6)	155 (5)
N2—H2*N*···O6^i^	0.87 (2)	2.00 (2)	2.857 (6)	171 (5)
C5—H5···O2^ii^	0.93	2.47	3.357 (7)	159
C14—H14···O4^iii^	0.93	2.53	3.457 (7)	175
C16—H16*A*···O1	0.93	2.46	3.207 (8)	138
C18—H18*B*···O2^iv^	0.96	2.53	3.339 (8)	142
C15—H15···*Cg*1^v^	0.93	2.72	3.629 (7)	167

Symmetry codes: (i) −*x*+1, −*y*+1, −*z*; (ii) *x*+1, *y*, *z*; (iii) −*x*, −*y*+3, −*z*; (iv) −*x*+1, −*y*+1, −*z*+1; (v) −*x*+1, −*y*+2, −*z*.
